# Novel and advanced MNP molecular markers accurately identify the genetic similarity of *Hypsizygus marmoreus* strains: a comparative evaluation with ISSR and antagonistic methods

**DOI:** 10.3389/ffunb.2025.1647126

**Published:** 2025-10-24

**Authors:** Honglei Zhao, Chuanzheng Wei, Tuo Zhang, Yihao Zhao, Xinyue Chen, Zhiwen Lv, Menghan Nie, Jian Li, Yuanzhen Liu, Baogui Xie, Xinrui Liu

**Affiliations:** ^1^ College of Horticulture, Fujian Agriculture and Forestry University, Fuzhou, Fujian, China; ^2^ Mycological Research Center, Fujian Agriculture and Forestry University, Fuzhou, Fujian, China

**Keywords:** genetic diversity, genetic similarity, *Hypsizygus marmoreus*, multiple nucleotide polymorphism, resequencing, strain differentiation

## Abstract

*Hypsizygus marmoreus* is a wood-rotting fungus of significant medicinal value, extensively cultivated industrially. As the scale of production expands, the issue of “same strains with different names” has become increasingly important, necessitating an accurate, efficient, and rapid method for variety identification. In this study, we resequenced 79 strains of *H. marmoreus* and selected 32 strains to construct a database comprised of 369 multiple nucleotide polymorphism (MNP) molecular markers and subsequently analyzed the genetic similarity among these strains. The results revealed that none of the 32 selected strains exhibited 100% genetic similarity. Specifically, the genetic similarity of the 369 MNP markers among the white strains ranged from 11.92% to 88.62%, while that of the gray strains ranged from 2.71% to 74.53%, indicating that the gray strains exhibited greater genetic diversity than their white counterparts. Furthermore, we compared the identification results of MNP molecular markers with those obtained from cross-plating experiments and ISSR molecular markers. This comparison highlighted the advantages of the MNP molecular marker method in terms of stability, accuracy, and high efficiency, thereby significantly contributing to the advancement of *H. marmoreus* strains identification and creation.

## 1 Introduction

*Hypsizygus marmoreus* (Peck) H.E. Bigelow possesses significant nutritional (Liu et al., 2011) and medicinal (ShangGuan, 2004) values and ranks among the principal artificially cultivated edible fungi in China. With the advancement of variety protection laws, the emphasis on intellectual property rights for edible fungi varieties has intensified. Consequently, an efficient, rapid, and precise method for variety identification has become critically important.

Given the close genetic relationships among many varieties, accurately and efficiently distinguishing between them poses a challenge. Additionally, the same variety often exhibits considerable diversity under varying cultivation conditions (Ling et al., 2022). Traditional morphological, cytological, and biochemical methods are prone to environmental influences and subjective interpretation (Worrall, 1997). In contrast, molecular markers based on DNA polymorphisms offer substantial advantages, including a greater number of markers and enhanced availability, effectively differentiating the genetic sequences of individual strains. Moreover, the identification of molecular markers remains unaffected by environmental variables or developmental stages. Several molecular methods, such as Simple Sequence Repeats (SSR) (Fu et al., 2010), Random Amplified Polymorphic DNA (RAPD) (Park et al., 1997; Sun and Lin, 2003), and Single Nucleotide Polymorphism markers (SNP), have gained widespread application.

ISSR (Inter-Simple Sequence Repeat) and RAPD methods rely on gel electrophoresis band patterns for identification. However, due to the randomness of primer annealing sites, PCR amplification often produces non-specific bands, resulting in poor experimental reproducibility (Oliveira and Azevedo, 2022). To improve reliability, researchers need to repeatedly optimize reaction systems, which increases experimental complexity and time costs (Lv et al., 2024). Additionally, the low-throughput nature of these methods (only capable of detecting a limited number of loci per run) and the subjective errors in manual band reading further limit their efficiency in large-scale applications (Zhao et al., 2017).

With the advancement of high-throughput sequencing technologies, SNP (Single Nucleotide Polymorphism) markers developed at the whole-genome level have gradually emerged as a new research trend due to their high reproducibility and precision (Li et al., 2010). Building upon this, researchers have further developed Multi-nucleotide Polymorphism (MNP) marker methods based on single nucleotide polymorphisms (Xu et al., 2020). Currently, MNP markers have been successfully employed for variety identification in rice, soybean, canola, eggplant, maize, tomato, and other crops. The fundamental principle involves multiple SNPs within the genome; examining a combination of alleles with distinct SNPs facilitates the differentiation of various individuals. Zhang et al. (2019) utilized 317 MNP markers to identify 12 varieties of *Artemisia argyi*, achieving 314 effective markers that successfully distinguished between *A. argyi* from different regions and identified varieties with a high degree of heterozygosity. Ling et al. (2022) screened 501 MNP marker sites based on the resequencing of 188 *Lentinula edodes* strains, designing primers to amplify the MNP markers through multiplex PCR. Similarly, Liu et al. (2023) constructed a database of MNP molecular markers for 163 strains of *Flammulina filiformi*s. This method is characterized by its accuracy, ease of operation, and cost-effectiveness.

Presently, China’s edible fungi industry is experiencing rapid development, and the selection and innovation of *H. marmoreus* varieties, an essential aspect, is marked by the troubling phenomenon of identical strains bearing different names. Therefore, the efficient and accurate identification of *H. marmoreus* varieties is paramount in the breeding process. In this study, we utilized 79 strains of *H. marmoreus* as starting materials. After resequencing and screening, we selected 32 strains to construct the MNP molecular markers database, which will aid in the identification of *H. marmoreus*. This represents a significant contribution to our edible fungi breeding efforts.

## 2 Materials and methods

### 2.1 Collection of samples and medium formula

A total of 79 strains (47 white and 32 gray) of *H. marmoreus* were used in this study, including 12 white cultivars and 3 gray cultivars cultivated in our laboratory. All of the above strains were preserved in the Fujian Provincial Center for the Preservation and Management of Edible and Medicinal Fungi Germplasm Resource (**Table 1**).

**Table 1:** Information on tested varieties.

Experiment number	Variety number	Variety source	Color
1	Hy0056	Tianjin	white
2	Hy0064	Dongying, Shandong	white
3	Hy0065	Dongying, Shandong	white
4	Hy0066	Beijing	white
5	Hy0067	Dongying, Shandong	white
6	Hy0068	Qinhuangdao, Hebei	white
7	Hy0069	Shanghai	white
8	Hy0070	Nanping, Fujian	white
9	Hy0071	Wuxi, Jiangsu	white
10	Hy0072	Zhangzhou, Fujian	white
11	Hy0073	Zhangzhou, Fujian	white
12	Hy0120	Shaoguan, Guangdong	white
13	Hy0121	Shaoguan, Guangdong	gray
14	Hy0122	Dongying, Shandong	gray
15	Hy0123	Maoming, Guangdong	gray
16	Hy0124	Shunchang, Fujian	white
17	Hy0125	Yongan, Fujian	white
18	Hy0126	Shanghai	white
19	Hy0127	Shanghai	gray
20	Hy0128	Zhangzhou, Fujian	gray
21	Hy0129	Lianyungang, Jiangsu	white
22	Hy0130	Lianyungang, Jiangsu	gray
23	Hy0131	Lianyungang, Jiangsu	white
24	Hy0132	Taixing, Jiangsu	white
25	Hy0133	Suqian, Jiangsu	gray
26	Hy0134	Zhangzhou, Fujian	white
27	Hy0135	Shunchang, Fujian	white
28	Hy0136	Cixi, Zhejiang	gray
29	Hy0137	Cixi, Zhejiang	white
30	Hy0138	Shunchang, Fujian	white
31	Hy0139	Zhangzhou, Fujian	white
32	Hy0140	Shanghai	white
33	Hy0141	Shanghai	gray
34	Hy0142	Kunshan, Jiangsu	white
35	Hy0143	Ningde, Fujian	white
36	Hy0144	Suqian, Jiangsu	white
37	Hy0145	Shanghai	gray
38	Hy0146	Shanghai	gray
39	Hy0147	Niigata, Japan	gray
40	Hy0148	Fukuoka, Japan	gray
41	Hy0149	Ibaraki, Japan	gray
42	Hy0150	Niigata, Japan	gray
43	Hy0151	Ibaraki, Japan	gray
44	Hy0152	Nagano, Japan	gray
45	Hy0153	Niigata, Japan	gray
46	Hy0154	Nagano, Japan	gray
47	Hy0155	Miyagi, Japan	gray
48	Hy0156	Toyama, Japan	gray
49	Hy0157	Toyama, Japan	gray
50	Hy0158	Nagano, Japan	gray
51	Hy0159	Nagano, Japan	gray
52	Hy0160	Nagano, Japan	gray
53	Hy0161	Nagano, Japan	gray
54	Hy0162	Niigata, Japan	gray
55	Hy0163	Ibaraki, Japan	gray
56	Hy0164	Hybrid strains	white
57	Hy0170	Zhangzhou, Fujian	white
58	Hy0171	Zhangzhou, Fujian	white
59	Hy0173	Zhangzhou, Fujian	white
60	Hy0185	Hybrid Strain	white
61	Hy0186	Hybrid Strain	white
62	Hy0187	Hybrid Strain	white
63	Hy0189	Hybrid Strain	white
64	Hy0190	Zhangzhou, Fujian	white
65	Hy0191	Zhangzhou, Fujian	white
66	Hy0204	Zhangzhou, Fujian	gray
67	Hy0205	Zhangzhou, Fujian	gray
68	Hy0206	Hybrid Strain	white
69	Hy0207	Hybrid Strain	white
70	Hy0208	Hybrid Strain	white
71	Hy0213	Hybrid Strain	gray
72	Hy0214	Hybrid Strain	gray
73	Hy0215	Hybrid strains	gray
74	Hy5031	Hybrid strains	white
75	Hy5055	Hybrid strains	white
76	Hy5091	Hybrid strains	white
77	Hm001	Zhangzhou, Fujian	white
78	Hm002	Zhangzhou, Fujian	white
79	Hm003	Hybrid Strain	white

Medium formula: PDA medium (potato 200g/L, glucose 20g/L, agar 20g/L, water).

### 2.2 DNA extraction and resequencing

Genomic DNA was extracted using a modified CTAB method (Lu, 2016). A 3 μL aliquot of DNA solution was mixed with 0.5 μL of 6× loading buffer and subjected to 1% agarose gel electrophoresis to assess DNA integrity. DNA samples passing quality control were sequenced on the Illumina NovaSeq 6000 platform using a paired-end 150 bp (PE150) library format, generating approximately 4 Gb of data per sample. The resequencing data for all *H. marmoreus* strains have been deposited in the Sequence Read Archive (SRA) database of NCBI under the accession number BioProject: PRJNA1332451.

The modified CTAB protocol proceeded as follows: first, transformants of *H. marmoreus* were inoculated into PDB medium and cultured statically at 28°C for 10 days before fresh mycelia were collected. Subsequently, approximately 100~200 mg of mycelia was placed in a 2 mL EP tube containing sterilized steel beads, to which 400 μL of SDEB buffer (100 mM NaCl, 50 mM EDTA, 0.25 M Tris-HCl, 5% SDS) was added. The samples were homogenized using a bead beater at 50 Hz for two cycles of 60 s each. Then, 400 μL of 2× CTAB buffer (2% CTAB, 100 mM Tris-HCl pH 8.0, 20 mM EDTA, 1.4 M NaCl, 1% PVP) was added and the mixture was vortexed thoroughly. This was followed by phenol-chloroform extraction with 250 μL of phenol and 250 μL of chloroform:isoamyl alcohol (24:1). After vigorous vortexing, the mixture was centrifuged at 13,000 rpm for 5 min at 4°C, and the supernatant was carefully collected. For DNA precipitation, a volume of isopropanol equivalent to 0.7 times that of the supernatant was added and mixed by gentle inversion. The DNA was pelleted by centrifugation at 13,000 rpm for 10 min at 4°C, after which the supernatant was discarded. The pellet was then resuspended in 900 μL of 70% ethanol and incubated at 4°C for 1 h. Following another centrifugation step under the same conditions, the supernatant was removed. Finally, the pellet was air-dried in a laminar flow hood to evaporate residual ethanol, dissolved in 100 μL of TE buffer containing RNase A, incubated at 55°C for 30 min, and stored at -20°C.

### 2.3 Population phylogenetic tree construction and sample screening

In this study, we used the maximum likelihood (ML) method to construct a population phylogenetic tree for the 79 strains collected. We used VCFtools software to filter the VCF files containing the results of SNP loci of the *H. marmoreus* population, then used vcf2phylip.py script (Subramanian et al., 2019) to convert the VCF files into phylip files, and used the GTRCAT model of RAxML software (Stamatakis, 2006), iterated 1000 times, and finally obtained the *H. marmoreus* population phylogenetic tree.

### 2.4 Resequencing population SNP variant site identification and quality control

The raw data were processed to filter out junctions and low-quality reads prior to population variant site detection. The clean data reads were aligned to the reference genome ‘HM62’ (GenBank assembly accession: GCA_013433165.1) using BWA software (version 0.7.17), and the results were converted to BAM format using SAMtools software (version 1.6) (Li, 2013; Petr et al., 2021). After marking PCR duplicates with the MarkDuplicates tool in GATK (version 4.2.0.0), variants were individually called for each sample using the HaplotypeCaller tool. The GVCF files of all samples were then merged to generate VCF files using the GenotypeGVCFs tool. The SNP and InDel VCF files were integrated using SelectVariants and further filtered and annotated with the following parameters: for SNPs, QD < 2.0, FS > 60.0, MQ < 40.0, SOR > 3.0, MQRankSum < -12.5; for InDels, QD < 2.0, FS > 200.0, SOR > 10.0. Finally, SNPs with a minor allele frequency (MAF) < 0.05 and a missing rate threshold > 0.2 were removed using a Python script (**Table 2**).

**Table 2:** The results of SNP quality control.

Filter condition	Number of SNPs
GATK VariantFiltration	3,175,345
MAF<0.05	1,926,536
SNP missing rate>20%	1,773,916
10<Site depth<2000	1,692,357

### 2.5 Screening of MNP molecular markers in *H. marmoreus*

Using the pysam module (Version 0.19.0; https://github.com/pysam-developers/pysam), we filtered SNP sites from VCF files preprocessed by VCFtools. The filtering criteria required: (1) sequencing depth >10× for each sample, (2) valid genotypes present in all samples, and (3) samples with identical genotypes not exceeding 95% of the total sample size, to obtain polymorphic SNP sites. Subsequently, we scanned the filtered SNPs using 100bp sliding windows, retaining windows containing 2–10 SNPs. We then calculated the polymorphism information content (PIC) for each window, and only selected regions meeting the following criteria: (1) PIC ≥ 0.5, and (2) adjacent window spacing >50kb. The final qualified window regions were designated as MNP markers.

### 2.6 Construction of MNP molecular markers library

Using python script to complete the genotype detection of the 32 *H. marmoreus* strains, the BAM file was used as input, and the count coverage function of the pysam module was used to calculate the number of reads at MNP markers, and 0.1 was used as the threshold for heterozygous genotypes to make a genotyping judgment. The obtained genotypes were stored in binary format using python’s pickle module (https://docs.python.org/3/library/pickle.html).

### 2.7 Calculation of genetic similarity of varieties in the library

The MNP molecular marker database contains the MNP genotype of each strain. By comparing the genotypes of two strains, the number of MNP markers with the same genotype n is counted, and the genetic similarity (GS) between the two strains is calculated, the formula of GS is: GS (%) = n/N×100%, N is the total number of MNP markers. Repeat the above process until the 32 strains have completed the two-by-two comparison.

### 2.8 Validation of cross-plating experiment

Approximately 20 mL of PDA medium was poured into 90 mm Petri dishes. After solidification, 5 mm mycelial plugs taken from the actively growing margins of each strain were inoculated onto the PDA plates at intervals of 2 cm. The plates were incubated at 25°C in darkness. Antagonistic reactions between strains were observed and photographed approximately 10 days after mycelial contact.

### 2.9 ISSR molecular marker validation

PCR amplification was carried out on 32 *H. marmoreus* strains using 20 ISSR primers selected from previously reported methods (Huang et al., 2009; Qiu, 2013). Each reaction was performed with three technical replicates. The primer sequences are listed in **Table 3**. The PCR reaction system was 25 μL: 12.5 μL Premix Taq, 1 μL template DNA, 2 μL primers (including the forward and reverse primers), and 9.5 μL sterile water. The amplification procedure was as follows: pre-denaturation at 94°C for 5min, denaturation at 94°C for 30s, annealing at 55°C for 30s, extension at 72°C for 1 min per kb, cycling 35 times and then extension at 72°C for 10min, and then stored at 4°C. PCR products were electrophoresed on a 1% agarose gel (120V, 80 mA, 30 min). After electrophoresis, the PCR products were manually counted for polymorphic bands, and clear bands were labeled as “1” and no bands were labeled as “0” to construct the 01 matrix. The Jaccard distance between the samples was calculated by using the pdist function of the scipy cluster hierarchy module (https://docs.scipy.org/doc/scipy/reference/cluster.html) in python, and the cluster tree was constructed by using the linkage function. Finally, the linkage function is used to construct the clustering tree.

**Table 3:** ISSR primer sequences.

Primer	Sequence (5’→3’)
P01	AGAGAGAGAGAGAGAGT
P02	GAGAGAGAGAGAGAGAT
P03	AGAGAGAGAGAGAGAGAYT
P04	GAGAGAGAGAGAGAGAAC
P05	TGCACACACACACAC
P06	GTGACACACACACAC
P07	GGATGCAACACACACACAC
P08	ACACACACACACACACCG
P09	GTGACGACTCTCTCTCTCT
P10	TCTCTCTCTCTCTCTCCG
P11	AAGAAGAAGAAGAAGAAGC
P12	TCTCTCTCTCTCTCTCA
P13	TCTCTCTCTCTCTCTCRT
P14	AGCAGCAGCAGCAGCAGCG
P15	MGTGTGTGTGTGTGT
P16	GGAGTGGTGGTGGTG
P17	AGTGTGTGTGTGTGT
P18	GTATGTATGTATGTATGG
P19	GTATGTATGTATGTATGC
P20	CCAGTGGTGGTGGTG

## 3 Results

### 3.1 SNP analysis and sample selection of *H. marmoreus* groups

**Figure 1:**
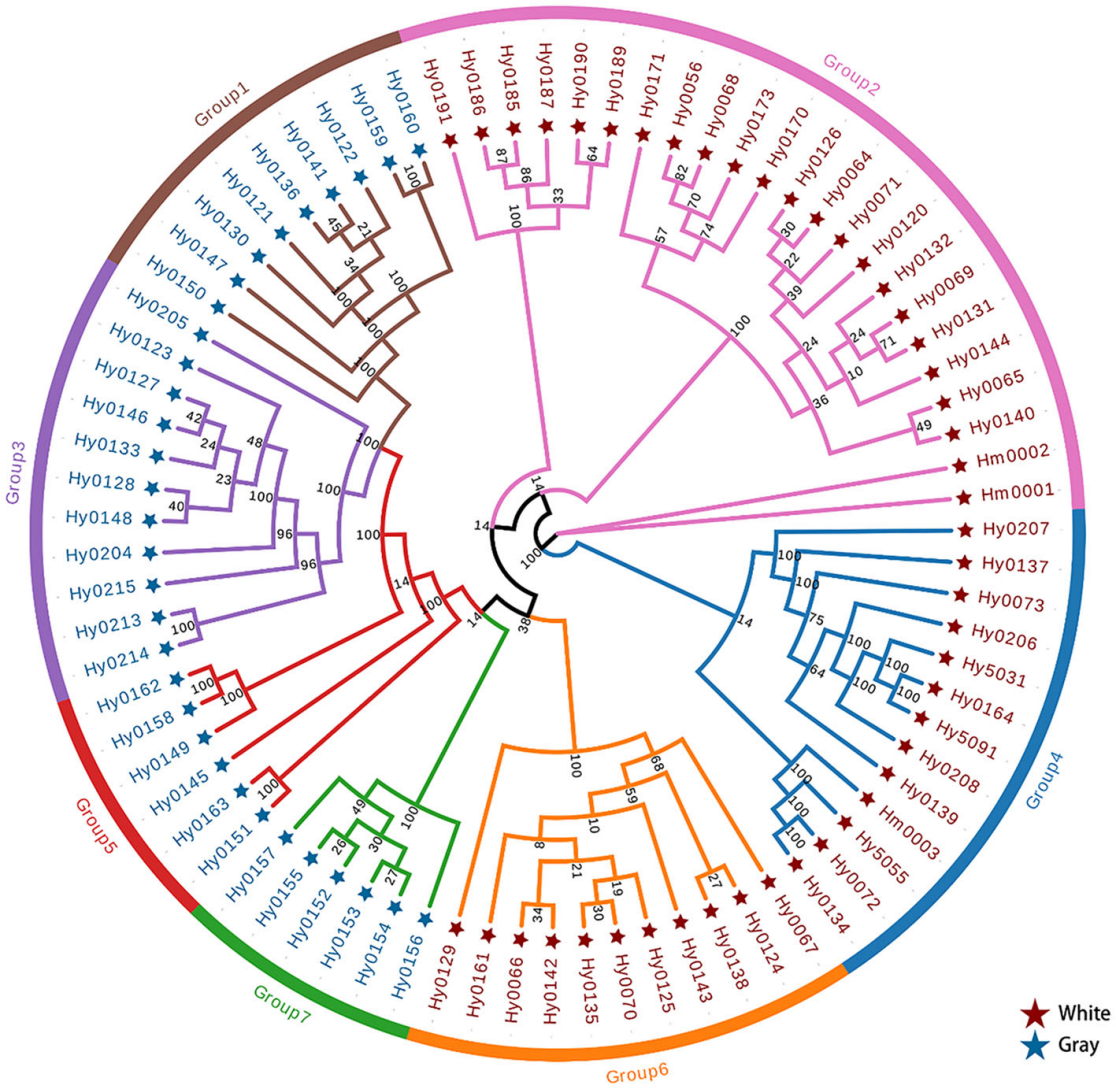
The phylogenetic tree of *H. marmoreus* population based on SNP sequences from 79 *H. marmoreus* strains.

We analyzed SNPs of *H. marmoreus* to construct a phylogenetic tree (**Figure 1**). The phylogenetic tree was constructed using sequences from all experimental strains of *H. marmoreus* to assess genetic distances and clustering patterns among the strains. The tree shown in **Figure 1** is unrooted and intended only to display relative genetic relationships. The first branch showed 100% bootstrap support and separated two clades composed of Branch A (comprising Group 2 and Group 4) and Branch B (comprising Group 1, Group 3, Group 5, Group 6, and Group 7). Branch A consisted predominantly of white strains, while Branch B primarily included gray strains, with the exception of Group 6.

The two groups within Branch A were comprised of white cultivars and hybrids, which clustered with but their separation only had 14% bootstrap support, which may have been caused by the insufficient sample population of *H. marmoreus* that we collected. According to the breeding process, we can know that part of Branch A is a hybrid or self-cross (Group 4) with Hy0072 and Hy0073 as parents, while the other part is a hybrid between the white cultivar Hy0056 and Group 6. Notably, Branch A does not exhibit a clear geographical distribution, as varieties from across China are represented. This phenomenon may be attributed to the factory cultivation of *H. marmoreus*, where propagation relies entirely on manual labor. Producers typically select varieties with superior traits, leading to a widespread distribution that tends to cluster within the same group.

Surprisingly, another group of white cultivars (Group 6) and gray strains were clustered in branch B. Moreover, Group 7 and Group 6 have a very close genetic distance, and it is hypothesized that these two groups may share a common ancestor, which is consistent with the conclusion that the white varieties originated from the gray varieties. Group 1, Group 3, and Group 5 may have been formed from the continued divergence of the ancestor of Group 7. We can see that there are some samples with extremely short genetic distances on the phylogenetic tree, indicating sample redundancy within the population. Therefore, we removed this portion of some samples displayed extremely short genetic distances within the phylogenetic tree, signifying redundancy within the population. Consequently, we eliminated these strains with closely related genetic distances, resulting in a refined collection of 32 strains, comprising 18 white and 14 gray strains (**Table 4**).

**Table 4:** *H.marmoreus* varieties of MNP marker library.

Experiment number	Variety number
1	Hm0001
2	Hm0002
3	Hm0003
4	Hy0056
5	Hy0070
6	Hy0072
7	Hy0073
8	Hy0123
9	Hy0130
10	Hy0145
11	Hy0147
12	Hy0149
13	Hy0150
14	Hy0151
15	Hy0152
16	Hy0158
17	Hy0159
18	Hy0164
19	Hy0185
20	Hy0186
21	Hy0187
22	Hy0190
23	Hy0205
24	Hy0206
25	Hy0207
26	Hy0208
27	Hy0213
28	Hy0214
29	Hy0215
30	Hy5031
31	Hy5055
32	Hy5091

### 3.2 MNP molecular marker database of *H. marmoreus*

**Figure 2:**
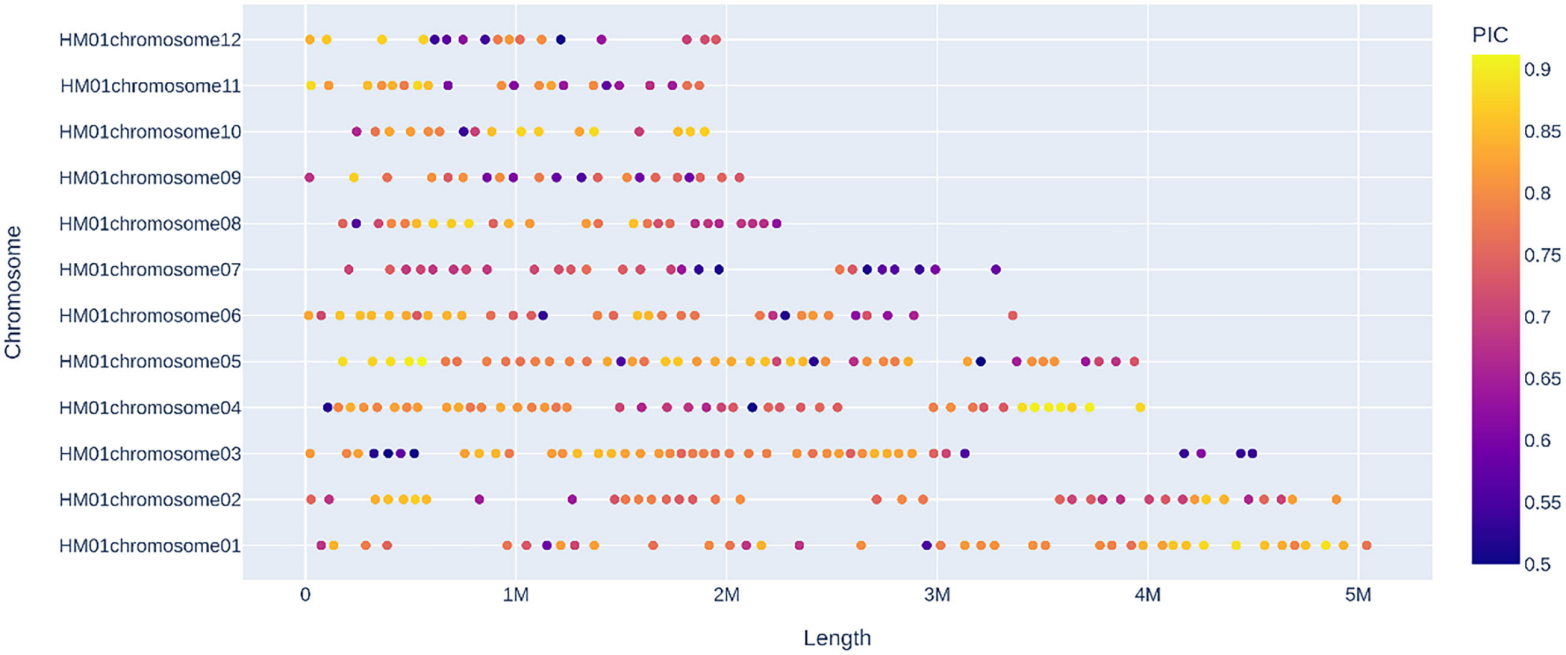
Distribution of MNP markers on chromosomes Sequencing depth >10×, number of SNPs ≥2 and ≤10, PIC ≥0.5, and distance between adjacent intervals >50kb.

32 *H. marmoreus* strains (18 white and 14 gray strains) were used to construct MNP molecular markers. The genome resequencing results of the 32 strains showed a comparison rate of 54.25% to 78.79% (average 67.22%) by bowtie2 and GATK analysis (**Table 5**). The variant sites among these strains were further examined and filtered out the InDel, and the SNP sites present between any two strains were obtained for the construction of MNP molecular markers in the next step. A total of 419,680 SNPs were found among the 32 *H. marmoreus* strains, and the SNP sites were evenly distributed on the chromosome, the density of SNP distribution was 1 SNP per 104 bases on average, except for chromosome02, where the density of SNP distribution was smaller, with 1 SNP per 192 bases on average (**Table 6**). The SNP sites were further filtered using VCFtools software with preset parameters, and 67,496 SNP sites were obtained. Based on these high-quality SNP sites, 369 MNP molecular markers were obtained by sliding window analysis using python script and the database was constructed (**Table A.1**). MNP markers were evenly distributed throughout the genome (**Figure 2**), containing a total of 1,231 SNP sites, with each MNP containing an average of 3.37 SNP sites. The PIC values of the MNP markers ranged from 0.50 to 0.91, with an average of 0.76, which was highly polymorphic.

**Table 5:** Resequencing results and comparison rate.

Variety number	Reads quantity	GC content (%)	Comparison rate (%)	Depth (%)
Hm0001	1797	48	70.86	95.79
Hm0002	1873	48	72.18	100.37
Hm0003	1673	47	67.41	88.41
Hy0056	2145	47	57.32	93.72
Hy0070	1685	47	78.79	88.02
Hy0072	1519	48	68.94	76.74
Hy0073	1674	48	68.50	83.07
Hy0123	1929	48	63.90	92.84
Hy0130	1546	48	57.10	74.08
Hy0145	1607	48	66.71	77.58
Hy0147	1804	48	65.56	90.51
Hy0149	1350	48	63.42	63.64
Hy0150	1479	48	64.91	72.41
Hy0151	1498	48	68.14	72.13
Hy0152	1675	47	72.42	85.03
Hy0158	1566	48	61.26	72.61
Hy0159	1464	47	54.25	66.91
Hy0164	2186	48	70.86	114.89
Hy0185	2193	48	75.11	121.67
Hy0186	2765	47	75.50	151.92
Hy0187	2434	48	74.70	133.84
Hy0190	2296	47	72.01	120.43
Hy0205	1445	48	61.40	72.03
Hy0206	1455	48	69.66	76.00
Hy0207	1595	48	69.55	87.49
Hy0208	1440	48	69.05	77.83
Hy0213	1651	48	64.06	85.94
Hy0214	1652	48	64.41	84.65
Hy0215	1333	48	63.47	68.06
Hy5031	1514	48	66.20	79.88
Hy5055	1455	47	68.05	76.59
Hy5091	1568	47	67.47	83.60

**Table 6:** Variants rate details of *H. marmoreus*.

Chromosome	Length	Variants	Variants rate
HM01chromosome01	5,516,215	50521	109
HM01chromosome02	4,999,619	25960	192
HM01chromosome03	4,517,060	45393	99
HM01chromosome04	4,177,578	55723	74
HM01chromosome05	4,102,088	41022	99
HM01chromosome06	3,364,172	41508	81
HM01chromosome07	3,318,489	30705	108
HM01chromosome08	2,643,500	35713	74
HM01chromosome09	2,421,378	26604	91
HM01chromosome10	2,260,060	24311	92
HM01chromosome11	2,188,314	19619	111
HM01chromosome12	2,080,224	15831	131

### 3.3 MNP sequencing depth analysis

**Figure 3:**
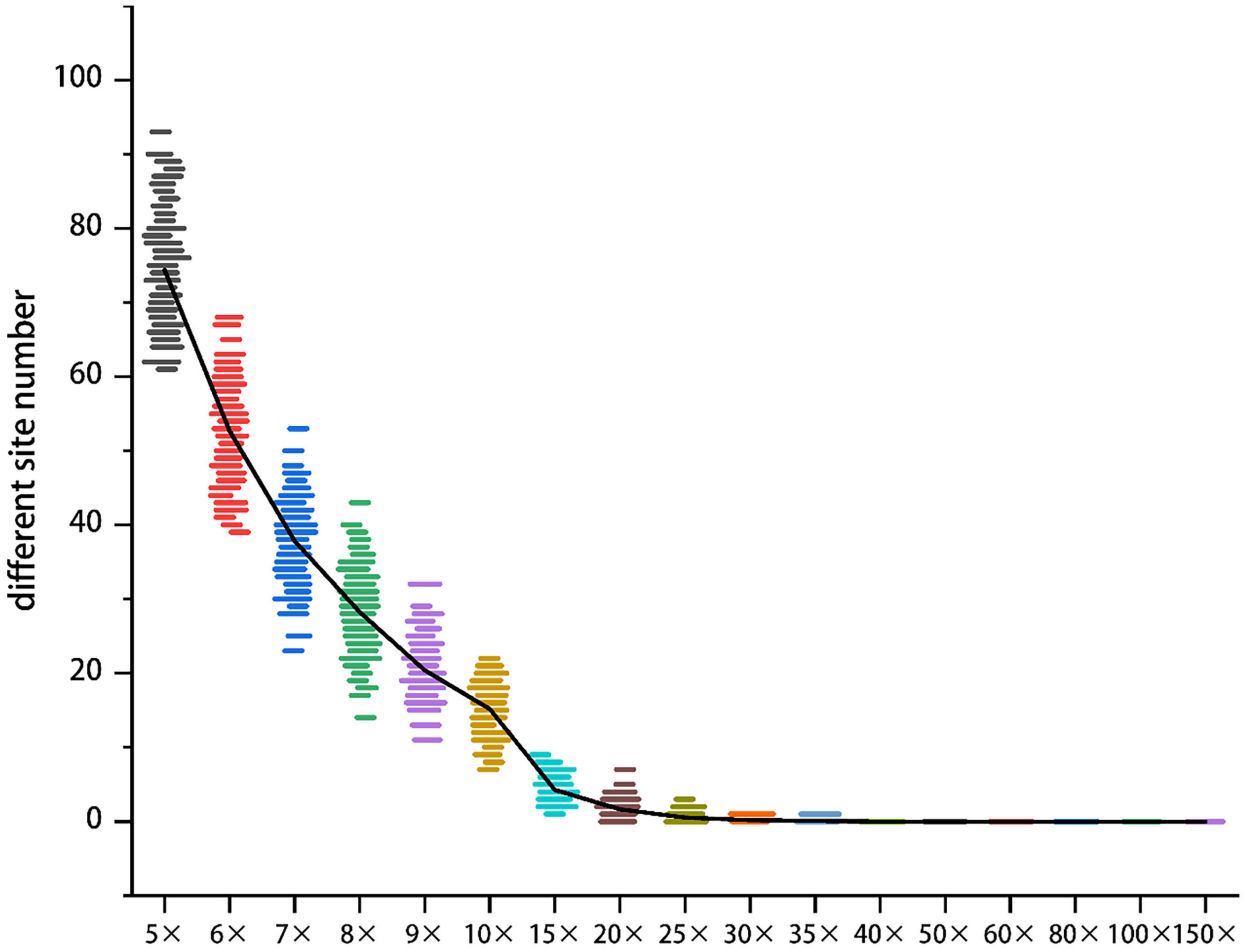
The effect of sequencing depth on the accuracy of MNP makers. The y-axis denotes the count of differential MNP loci, and the x-axis represents sequencing depth (color-coded). Beyond 40× depth, differential loci counts asymptotically approached zero.

The sequencing depth directly influences both the accuracy and cost of the MNP method. The genomes of edible fungi are typically small, allowing for a high sequencing depth to be achieved with a relatively low volume of sequencing data. In the case of *H. marmoreus*, a sequencing depth of 60× can be attained with a data volume of just 4 G. Consequently, we selected Hm0002, which has a sequencing data volume of 8 G, as our experimental sample. We utilized GATK software to simulate various MNP site scenarios and to quantify the number of differential MNP sites (**Figure 3**).

As the sequencing depth increases, the number of differential sites gradually declines, and the data distribution becomes more concentrated. Once the sequencing depth reaches 35×, the number of differential sites approaches zero. At a depth of 40×, the number of differential sites remains at zero, while the cost continues to rise without a significant improvement in accuracy. This finding suggests that a sequencing depth of 40× is sufficient to ensure the accuracy of MNP.

### 3.4 MNP markers to analyze the genetic similarity among *H. marmoreus* strains

**Figure 4:**
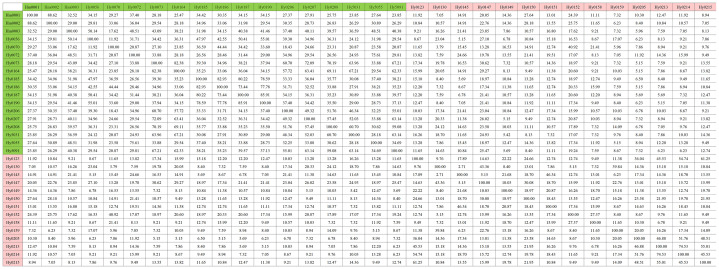
Genetic similarity matrix of strains in the MNP marker library. White strains are filled with light green, gray strains are filled with light red.

Using the previously constructed MNP markers, the genetic similarity (GS value) among 32 *H. marmoreus* strains was calculated. The results showed that no genetically identical strains existed among the 32 strains involved in this study, and the genetic similarity between individuals of the same strain was 100%. The highest genetic similarity was observed between strains Hm001 and Hm002 (88.62%), while the lowest was between the gray strains Hy0130 and Hy0145 (2.71%). Additionally, the genetic similarity among white *H. marmoreus* strains ranged from 11.92% to 88.62% (with an average of 39.64%), while among gray strains, it ranged from 2.71% to 74.53% (**Figure 4**). Among the gray strains, Hy0213, Hy0214, and Hy0215 were derived from hybridization between parental strains Hy0123 and Hy0205. The genetic similarities between Hy0213 and Hy0214, Hy0213 and Hy0215, and Hy0214 and Hy0215 were 74.53%, 55.01%, and 45.53%, respectively. This indicates that the genetic diversity of gray *H. marmoreus* strains is higher than that of white strains.

**Figure 5:**
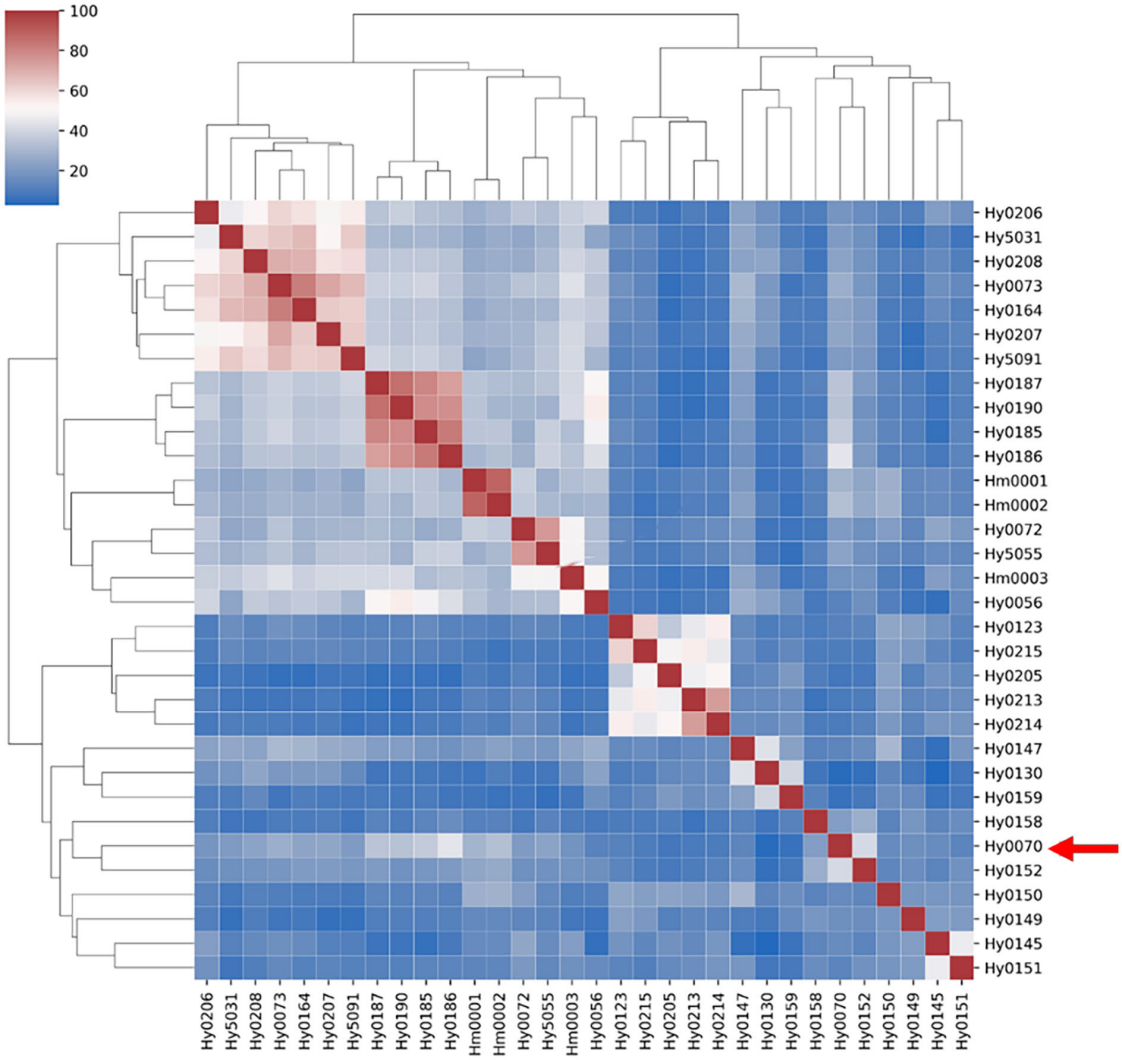
Clustering heatmap of strains of MNP marker library. Red arrows point to white strain Hy0070.

Cluster analysis based on the genetic similarity values calculated from the MNP markers revealed that the 32 *H. marmoreus* strains were clearly divided into two major groups: white and gray. However, the white strain Hy0070 was exceptionally clustered within the gray strain group (**Figure 5**).

### 3.5 Comparison of MNP molecular markers with cross-plating experiments

**Figure 6:**
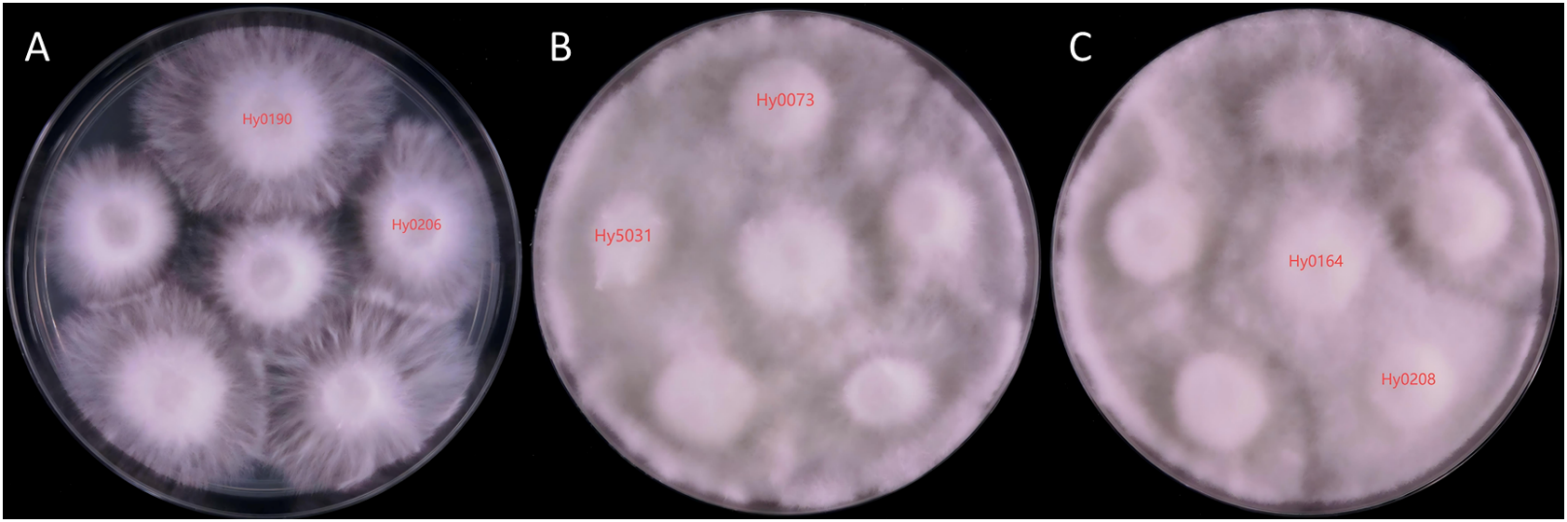
Mycelium antagonistic reactions between different strains of *H. marmoreus*. **(A)** Mycelial antagonism assay plate photograph of Hy0190 and Hy0260, showing a clear antagonistic zone line between the two strains, indicating significant antagonistic reaction. **(B)** Mycelial antagonism assay plate photograph of Hy5031 and Hy0073, showing no distinct antagonistic zone line between the two strains, indicating weak antagonistic reaction. **(C)** Mycelial antagonism assay plate photograph of Hy0164 and Hy0208, showing no antagonistic zone line between the two strains, indicating no antagonistic reaction.

The 32 *H. marmoreus* strains provided for testing underwent antagonism experiments. As these strains were selectively screened, none exhibited 100% genetic similarity. By documenting the results and comparing them with the MNP molecular marker findings, we discovered that the genetic similarity of the MNP markers between strains exhibiting significant antagonistic interactions was less than 76%. Their genetic similarity (GS) values ranged from 6.23% to 75.61%, exemplified by the pronounced antagonistic relationship between Hy0190 and Hy0206 (**Figure 6A**).

The cases where antagonism could not accurately differentiate the strains were categorized into two types. The first type included Hy0073 and Hy5031 (**Figure 6B**), which demonstrated an ambiguous antagonistic line and lacked a significant antagonistic response, yielding a GS value of 27.10%. The second type comprised Hy0164 and Hy0208 (**Figure 6C**), which exhibited no antagonistic response whatsoever, with a GS value of 69.11%. This indicates that antagonistic reactions alone cannot fully distinguish between the two strains.

### 3.6 Comparison of MNP molecular marker method with ISSR marker method

**Figure 7:**
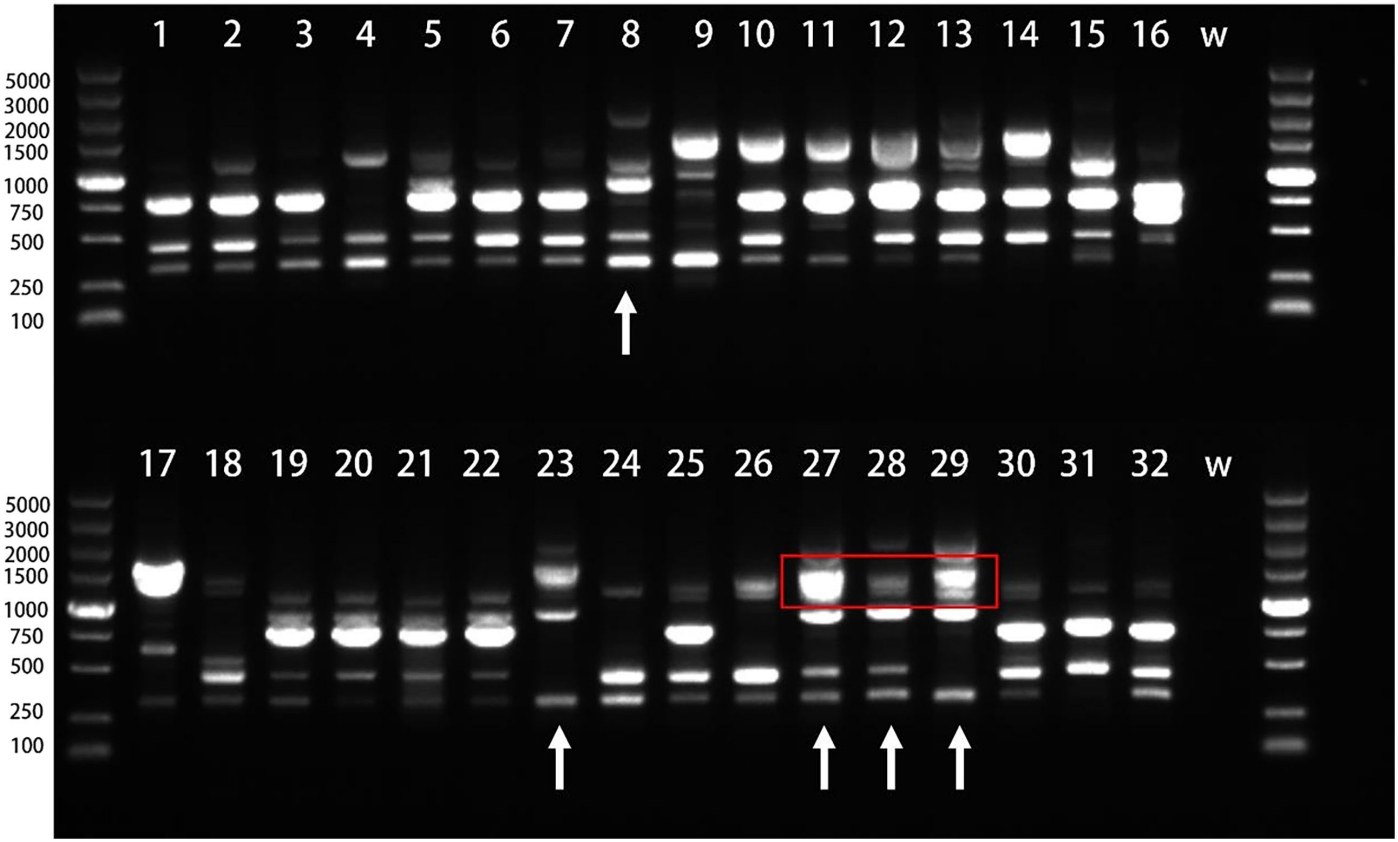
Electrophoresis of PCR products amplified by primer P05. The number is the experiment number in **Table 4**, W stands for H2O;the red box highlights the region in lanes 27–29 where the progeny exhibit a coalesced, thick band. This is due to the inheritance of two closely-sized parental bands (from lanes 8 and 23, within the 1000–1500 bp range) that are not resolvable as distinct fragments in the progeny under these experimental conditions.

We employed 20 ISSR primers for PCR amplification of 32 *H. marmoreus* strains and identified 10 primers (P03, P04, P05, P06, P07, P08, P11, P13, P14, P17) exhibiting strong repeatability and high polymorphism. Utilizing these 10 primers, we amplified a total of 62 polymorphic bands, averaging 6.2 bands per primer, with P05 illustrated in **Figure 7**.

Among the strains, Hy0123 (lane 8) and Hy0205 (lane 23) were crossed to yield Hy0213 (lane 27), Hy0214 (lane 28), and Hy0215 (lane 29). The significant divergence in band types between Hy0123 and Hy0205 suggests that the offspring may inherit bands from both parental strains while potentially gaining or losing some bands, thereby producing novel bands. Consequently, the hybrids can be distinguished from their progenitors via ISSR analysis. However, as highlighted in the red box of the figure, lanes 8 and 23 display two bands of similar sizes, approximately 1000~1500 bp, which appear as indistinguishable thick bands in their progeny (lanes 27, 28, and 29). Additionally, the hybrids exhibited fewer polymorphic bands, indicating a need for more ISSR primers to effectively differentiate hybrids derived from the same parents.

**Figure 8:**
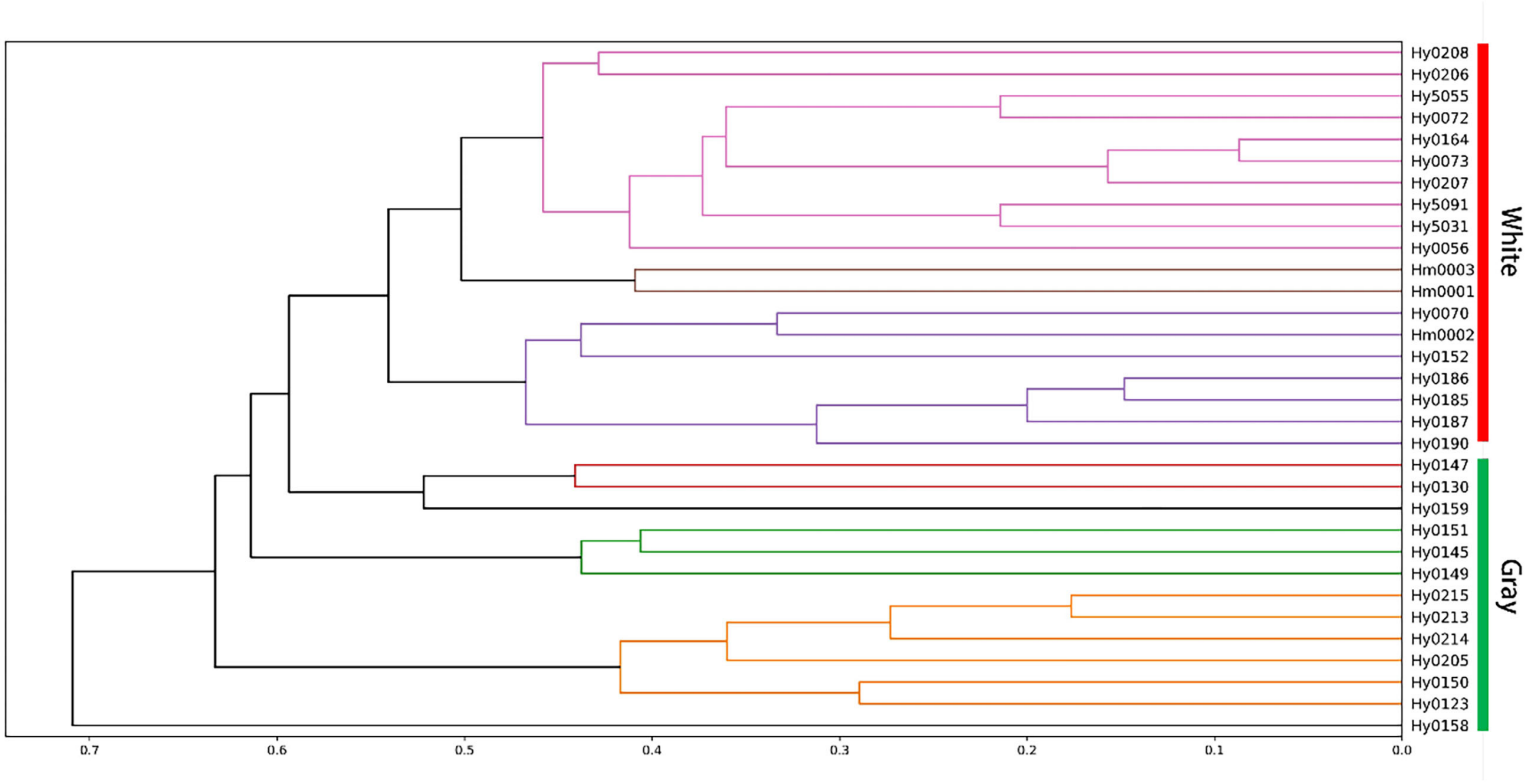
ISSR clustering map of *H. marmoreus* strains.

The polymorphic bands were subjected to clustering analysis, resulting in the generation of a clustering tree (**Figure 8)**. The findings revealed that the 32 strains were classified into seven distinct categories, clearly differentiated by color. The white strains were grouped into three categories, while the gray strains formed four. No strains with identical genetic coefficients were detected, confirming the distinctiveness of the 32 strains, consistent with the assessments made using the MNP marker method. In contrast to the clustering heatmap results from the MNP marker database, ISSR markers failed to cluster strain Hy0070 within the brown variety branch. This discrepancy may stem from inherent systematic errors in ISSR technology or the insufficient number of polymorphic bands employed, resulting in constrained resolution for population phylogenetic analysis.

## 4 Discussion

In this study, no instances of genetic similarity exceeding 90% but below 100% were observed. Given that *H. marmoreus* primarily undergoes asexual reproduction in practical applications with an extremely low mutation rate, we referred to the threshold range specified in the “MNP Marker Method for Plant Variety Identification” and established stricter criteria: varieties with a genetic similarity (GS) ≥ 99% were defined as highly similar or identical varieties, while those with GS < 99% were classified as distinct varieties. No intermediate category of “similar varieties” was established.

Elucidating the population evolutionary relationships of *H. marmoreus* is of significant importance for guiding breeding strategies. Using whole-genome resequencing technology, we conducted a phylogenetic analysis of 79 *H. marmoreus* strains, dividing them into seven major groups. This number of groups differs from the four major groups reported by Gong et al. (2019) based on resequencing of 38 germplasms, likely reflecting the expanded sample size and genetic diversity in our study. Although the widespread presence of hybrid varieties somewhat influenced the clustering structure, the division of core groups remained stable.

Notably, the inclusion of Japanese white strains in Group 6 provides clues to their origin. This finding aligns with historical practices during the Meiji era in Japan, which involved the systematic introduction of edible fungal germplasms from the Asian mainland. The sharing of mitochondrial-specific mutation sites between Group 1 and Group 6 further supports the effectiveness of using molecular markers such as mitochondrial DNA to trace the origins of edible fungi (Liu, 2008).

The phylogenetic topology revealed that Group 6 forms a clade with four gray variety groups (Groups 1, 3, 5, and 7). Professor Zhang Jisen’s research team (Wang et al., 2021), through resequencing analysis of 92 strains, also proposed that *H. marmoreus* comprises four main subpopulations. They explicitly stated that white varieties are albino mutants of gray strains rather than independently originated, providing genome-wide evidence supporting the possibility of a gray ancestor. Particularly noteworthy is the closest genetic distance between Group 7 and Group 6, which offers direct evidence for the hypothesis that white varieties diverged from a gray ancestor (Wang et al., 2021). The similarity of mitochondrial sequences between some strains in Group 4 and gray varieties suggests the existence of intraspecific gene flow, consistent with reports of rich genetic diversity among *H. marmoreus* strains (Liu, 2008; Qiu, 2013).

Based on these findings, we propose that all *H. marmoreus* varieties likely originated from a common ancestor (possibly even a single original strain), and this ancestor was most probably a gray variety. This complex genetic background may increase the difficulty of variety identification within this population.

In this experiment, we utilized 32 potentially divergent strains of *H. marmoreus* as materials and developed MNP molecular markers using identified SNP markers. A total of 369 MNP markers were obtained, encompassing 1,231 SNPs, of which 55.4% were situated in the exon region. This suggests that variation within the exon region may contribute significantly to population polymorphisms. Although whole-genome SNPs contain more variant information, some represent false positives due to the biases inherent in the high-throughput sequencing process and errors from sequence comparison software. Accuracy is paramount for variety identification; for instance, Peng et al. (2018) designed 2,029 MNP markers in rice (with a genome size of approximately 400 Mb). In comparison, we contend that 369 markers are sufficient and reliable for *H. marmoreus* (with a genome size of about 42 Mb).

**Figure 9:**
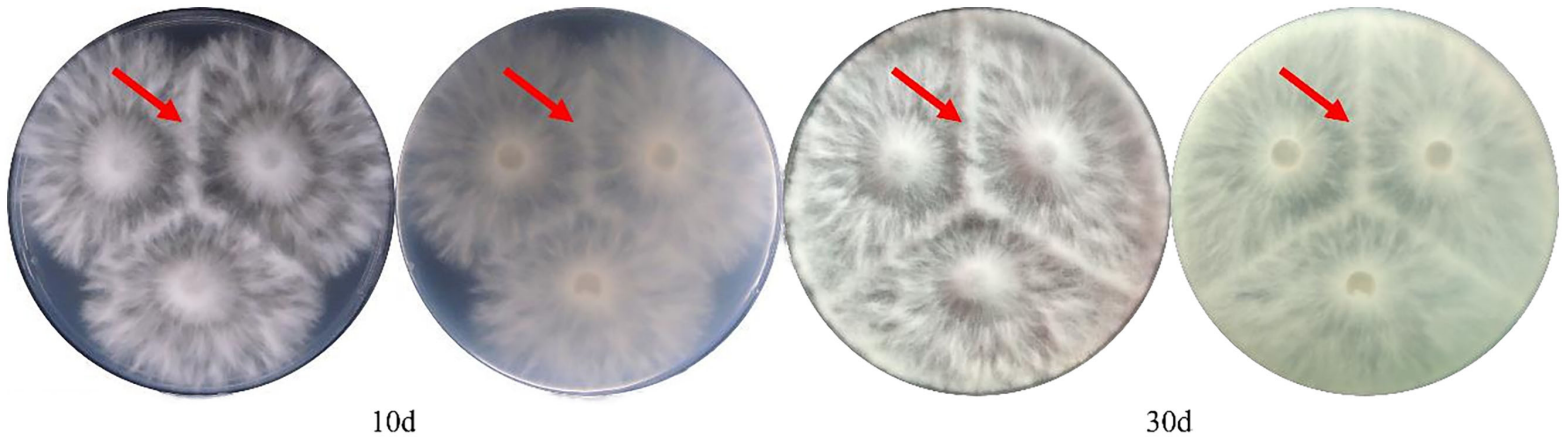
Antagonistic response of Hy0123 self-confrontation at 10d and 30d.

During the experimental process, we employed cross-plating experiments and the ISSR molecular marker method to validate the MNP molecular markers developed in this study. Most strains used for validation yielded identification results consistent with those of the MNP molecular markers. However, in the confrontation cross-plating experiments between strains, it proved challenging to maintain strict control over culture conditions and strain viability, which influenced the outcomes. Consequently, the same strain may exhibit different results under varying inoculations or environmental conditions. The antagonistic response, assessed through the confrontation of mycelium from different strains during growth, aims to discern varietal differences. Nonetheless, numerous instances of subtle antagonism were observed, potentially attributable to weak antagonistic responses between varieties or the entanglement of dense and sparse mycelium. Notably, some varieties, such as Hy0123 in this experiment (**Figure 9**), produced discernible antagonistic lines upon confrontation. Thus, while the results from the cross-plating experiment aligned with those obtained through MNP molecular markers, certain varieties remained indistinguishable via antagonism, whereas MNP markers could accurately differentiate and identify them. Furthermore, some types of antagonistic responses among *H. marmoreus* varieties are not easily discernible to the naked eye and are inherently subjective. The increase in varietal diversity can lead to a substantial rise in workload, suggesting that antagonistic responses possess inherent limitations in identifying *H. marmoreus* varieties.

Subsequently, during ISSR validation, we observed that the gel electrophoresis bands for the same pair of primers could differ or appear blurred when the same template underwent multiple PCR amplifications. Zhao (2014) conducted four repetitions of primers for the same ISSR molecular marker on a single apricot mushroom strain, revealing differences in the bands amplified from the same strain across different batches. Potential instability may arise when using ISSR markers to differentiate between *H. marmoreus* varieties.

In this study, the MNP marker method serves as an identification technique specifically designed to distinguish different varieties within the *H. marmoreus* group and cannot be employed to differentiate among various edible fungi varieties.

Although we employed the fundamental principles of the Plant Variety Identification MNP Marker Method in constructing the MNP marker database for *H. marmoreus*, our study was primarily based on resequencing rather than the multiplex PCR method, given the small genome size of edible fungi. The primary process of the multiplex PCR MNP method involves designing primer kits, amplifying the samples under examination alongside control samples, sequencing the amplification products, and analyzing genetic similarity. In contrast, the MNP method utilized in this study focuses on sequencing the DNA of interest and analyzing its genetic similarity with known varieties. This approach offers several advantages: the MNP molecular marker library can be expanded upon the identification of new varieties, the method based on second-generation sequencing technology facilitates more rapid and convenient detection results, and the MNP marker positions screened from high-polymorphism SNP clusters exhibit greater polymorphism (Fu, 2016; Li, 2020). For the established MNP molecular marker library, each time a new strain is identified, its MNP genotype can be updated in the library, eliminating the need for control samples and allowing for a comprehensive analysis against all known varieties. This significantly reduces the workload of strain identification, yielding more scientific and objective results. While the establishment of the MNP database requires specialized knowledge and equipment, which poses challenges for factories, enterprises, and edible fungi enthusiasts, the resequencing technology employed in this study has been widely adopted without technical barriers.

The results of this study demonstrate that the MNP marker method can avoid error-prone sequences such as simple repeats and consecutive base mutations when screening target regions. Meanwhile, the high-throughput sequencing-based MNP markers reduce the uncertainties introduced by multiple PCR amplification cycles, thereby significantly improving reproducibility and accuracy. This efficient, convenient, and precise capability for variety identification and authentication not only facilitates the improvement and innovation of new edible fungi varieties but also plays a crucial role in advancing the edible fungi industry and its commercial development.

## 5 Conclusions

This study conducted whole-genome resequencing of 79 *H. marmoreus* varieties and selected 32 representative strains to establish a database containing 369 MNP markers. Genetic analysis revealed no completely identical strains among the selected varieties, with white strains showing 11.92%~88.62% genetic similarity and brown strains demonstrating 2.71%~74.53%, indicating higher genetic diversity in brown strains. Compared to antagonism tests and ISSR markers, the MNP marker technique exhibited superior stability, accuracy, and operational efficiency.

## Data Availability

The datasets presented in this study can be found in online repositories. The names of the repository/repositories and accession number(s) can be found below: https://www.ncbi.nlm.nih.gov/, PRJNA1332451.

## References

[B1] Fu L. X. (2016). SNP genotyping using low-depth resequencing data and its application in brassica vegetables (Beijing: Chinese Academy of Agricultural Sciences).

[B2] Fu L. Z. Zhang H. Y. Wu X. Q. Li H. B. Wei H. L. Wu Q. Q.. (2010). Evaluation of genetic diversity in *Lentinula edodes* strains using RAPD, ISSR and SRAP markers. World J. Microbiol. Biotechnol. 26, 709–716. doi: 10.1007/s11274-009-0227-8

[B3] Gong M. Wu Y. Y. Li Y. Yang H. R. Wang Y. Wan J. N.. (2019). “Genome-wide resequencing of *hypsizygus marmoreus* and GWAS analysis of growth rate,” (Colorful Fungi, Beautiful China—Proceedings of the 2019 Annual Conference of the China Society of Mycology).

[B4] Huang C. Y. Li H. P. Zhang J. X. Chen Q. (2009). Verification of genuineness for edible mushroom spawn-ISSR (China: Agricultural Industry Standard).

[B5] Li B. J. Huang C. Q. Chen C. S. Lan C. Z. Chen Q. H. Weng Q. Y. (2010). Comparison and Improvement of Two Detection Methods for SSR Amplification products in the late blight pathogen genome. Biotechnol. Bull 11, 6. doi: CNKI:SUN:SWJT.0.2010-11-035

[B6] Li H. (2013). Aligning sequence reads, clone sequences and assembly contigs with BWA-M EM. arXiv preprint arXiv 1303, 3997. doi: 10.48550/arXiv.1303.399

[B7] Li J. D. (2020). Changes and developments in the international plant variety protection system and China’s response. Intellect Property Rights 227, 59–71.

[B8] Ling Y. Y. Zhang M. Z. Ling Z. Y. Cao B. Wu X. P. Peng H.. (2022). Evolutionary relationship and a novel method of efficient identification of *Lentinula edodes* cultivars in China. Mycosphere. 13, 56–85. doi: 10.5943/mycosphere/si/1f/3

[B9] Liu L. (2008). Study on Classification Methods and Genetic Diversity of Hypsizygus marmoreus. Master's thesis. (Qingdao City, Shandong Province, China: Qingdao Agricultural University).

[B10] Liu Y. Chen H. Feng Z. Y. Chen J. M. Wang H. Yu H. L.. (2011). Analysis on correlation between LBL evaluation and agronomic characters of Hypsizygus marmoreus. Shanghai J. Agric. 27, 116–120. doi: 10.3969/j.issn.1000-3924.2011.02.027

[B11] Liu F. Wang S. H. Jia D. H. Tan H. Wang B. Zhao R. L. (2023). Development of multiple nucleotide polymorphism molecular markers for Flammulina filiformis cultivars identification. J. Fungi 9, 330. doi: 10.3390/jof9030330, PMID: 36983498 PMC10056640

[B12] Lu Y. P. (2016). Adenylate cyclase regulation of mycelial growth and ascospore formation in Flammulina filiformis (Fuzhou: Fujian Agriculture and Forestry University).

[B13] Lv Z. W. Yang H. Wei C. Z. Ye X. Y. He S. Y. Tao Y. X.. (2024). Establishment of identification techniques for MNP molecularly marker strains of Flammulina filiformis. J. Mycol 43, 22–38. doi: 10.13346/j.mycosystema.230156

[B14] Oliveira M. Azevedo L. (2022). Molecular markers: an overview of data published for fungi over the last ten years. J. Fungi 8, 803. doi: 10.3390/jof8080803, PMID: 36012792 PMC9410331

[B15] Park W. M. Ko H. G. Park R. J. Hong K. S. (1997). Differentiation of *Lentinus edodes* isolates in Korea by isozyme polymorphisms and random amplified polymorphic DNA (RAPD) analysis. Korean J. Mycol 25, 176–190.

[B16] Peng H. Zhang J. Chen H. Zhang J. (2018). A method for testing substantial derivation relationship of plant varieties. Patent Number: CN201510148657.7. Jianghan University; Center for Science and Technology Development, Ministry of Agriculture.

[B17] Petr D. James K. B. Jennifer L. John M. Valeriu O. Martin O. P.. (2021). Twelve years of SAMtools and BCFtools. Gigascience 10, giab008. doi: 10.1093/gigascience/giab008, PMID: 33590861 PMC7931819

[B18] Qiu C. S. (2013). Cultivated traits and molecular markers in genetic diversity study of *Hypsizygus marmoreus* in China (Guang Zhou: South China University of Technology).

[B19] ShangGuan Z. J. (2004). Biological characteristics and cultivation technology of Hypsizygus marmoreus. Edible fungi 26, 16–18. doi: 10.3969/j.issn.1000-8357.2004.01.012

[B20] Stamatakis A. (2006). RAxML-VI-HPC: maximum likelihood-based phylogenetic analyses with thousands of taxa and mixed models. Bioinformatics 22, 2688–2690. doi: 10.1093/bioinformatics/btl446, PMID: 16928733

[B21] Subramanian S. Ramasamy U. Chen D. (2019). VCF2PopTree: a client-side software to cons truct population phylogeny from genome-wide SNPs. PeerJ 7, e8213. doi: 10.7717/peerj.8213, PMID: 31824783 PMC6901002

[B22] Sun Y. Lin F. C. (2003). Analysis of genetic diversity in natural germplasm of *Lentinula edodes* in China using RAPD technique. Mycosystema. 22, 387–393. doi: 10.1023/A:1022289509702

[B23] Wang G. Chen L. Tang W. Wang Y. Zhang Q. Wang H.. (2021). Identifying a melanogenesis-related candidate gene by a high-quality genome assembly and population diversity analysis in Hypsizygus marmoreus. J. Genet. Genomics 48, 13. doi: 10.1016/j.jgg.2021.01.002, PMID: 33744162

[B24] Worrall J. J. (1997). Somatic incompatibility in basidiomycetes. Mycologia. 89, 24–36. doi: 10.1080/00275514.1997.12026751

[B25] Xu Y. B. Yang Q. N. Zheng H. J. Xu Y. F. Sang Z. Q. Guo Z. F.. (2020). Genotyping by target sequencing (GBTS) and its applications. Scientia Agricult Sinica 53, 6–27. doi: 10.3864/j.issn.0578-1752.2020.15.001

[B26] Zhang Y. Peng H. Dong Y. H. Li T. T. Fang Z. C. Zhu S. X.. (2019). Application of an accurate identification technology MNP marker method in the identification of Qiai. China Assoc. Acupunct Moxibustion, 1609–1615.

[B27] Zhao F. (2014). Varietal identification of commercially cultivated strains of *Lentinula edodes*, *Auricularia polytricha*, *Agrocybe aegerita*, *Pleurotus pulmonarius* and *Agaricus bisporus* in Fujian Province (Fuzhou: Fujian Agriculture and Forestry University).

[B28] Zhao Y. N. Wang Y. Zhang D. J. (2017). Establishment and optimization of SSR-PCR reaction system in mung bean. Jiangsu Agric. Sci. 45, 4. doi: 10.15889/j.issn.1002-1302.2017.08.006

